# Secreted mucins in pseudomyxoma peritonei: pathophysiological significance and potential therapeutic prospects

**DOI:** 10.1186/1750-1172-9-71

**Published:** 2014-05-05

**Authors:** Afshin Amini, Samar Masoumi-Moghaddam, Anahid Ehteda, David Lawson Morris

**Affiliations:** 1 Department of Surgery, St George Hospital, The University of New South Wales, Level 3, Clinical Sciences (WR Pitney) Building, Gray Street, Kogarah, Sydney, NSW 2217, Australia

**Keywords:** Pseudomyxoma peritonei, PMP, Mucin, MUC2, Goblet cells, Appendix

## Abstract

Pseudomyxoma peritonei (PMP, ORPHA26790) is a clinical syndrome characterized by progressive dissemination of mucinous tumors and mucinous ascites in the abdomen and pelvis. PMP is a rare disease with an estimated incidence of 1–2 out of a million. Clinically, PMP usually presents with a variety of unspecific signs and symptoms, including abdominal pain and distention, ascites or even bowel obstruction. It is also diagnosed incidentally at surgical or non-surgical investigations of the abdominopelvic viscera. PMP is a neoplastic disease originating from a primary mucinous tumor of the appendix with a distinctive pattern of the peritoneal spread. Computed tomography and histopathology are the most reliable diagnostic modalities. The differential diagnosis of the disease includes secondary peritoneal carcinomatoses and some rare peritoneal conditions. Optimal elimination of mucin and the mucin-secreting tumor comprises the current standard of care for PMP offered in specialized centers as visceral resections and peritonectomy combined with intraperitoneal chemotherapy. This multidisciplinary approach has reportedly provided a median survival rate of 16.3 years, a median progression-free survival rate of 8.2 years and 10- and 15-year survival rates of 63% and 59%, respectively. Despite its indolent, bland nature as a neoplasm, PMP is a debilitating condition that severely impacts quality of life. It tends to be diagnosed at advanced stages and frequently recurs after treatment. Being ignored in research, however, PMP remains a challenging, enigmatic entity. Clinicopathological features of the PMP syndrome and its morbid complications closely correspond with the multifocal distribution of the secreted mucin collections and mucin-secreting implants. Novel strategies are thus required to facilitate macroscopic, as well as microscopic, elimination of mucin and its source as the key components of the disease. In this regard, MUC2, MUC5AC and MUC5B have been found as the secreted mucins of relevance in PMP. Development of mucin-targeted therapies could be a promising avenue for future research which is addressed in this article.

## Introduction

With an estimated incidence of 1–2 out of a million [1], pseudomyxoma peritonei (PMP, ORPHA26790)-also known as adenomucinosis or gelatinous ascites- is listed as a rare disease by the NIH Office of Rare Diseases Research (ORDR) and National Organization for Rare Disorders (NORD). As an indolent neoplasm with unspecific manifestations, PMP tends to be misdiagnosed, or discovered at advanced stages. Moreover, it is a challenging entity with debilitating, even fatal complications. Despite a multidisciplinary approach composed of an extensive surgical procedure and chemotherapy, PMP frequently recurs and increasingly jeopardizes quality of life. Being ignored in research, however, PMP remains poorly understood and enigmatic. This is despite the fact that “*rare diseases are rare, but rare disease patients are numerous”*[2] and those with progressive, life-threatening courses deserve to be further explored. To enhance outcomes of the conventional therapy, novel approaches based on in-depth understanding of the pathological processes and biological events in the pathogenesis of the disease are warranted. Since PMP and mucin are inextricably linked, any therapeutic intervention needs to properly target the mucin ectopy. In this article, the current knowledge on the crucial role of mucin in the pathogenesis of the disease is reviewed and a number of potential therapeutic strategies for mucin elimination in PMP are addressed.

### Definition

PMP is characterized by dissemination of mucinous tumor implants on peritoneal surfaces and progressive accumulation of mucinous ascites throughout the peritoneal cavity resulting in the so-called “jelly belly”.

### Etiology

Since initial descriptions of PMP as a syndrome in association with an ovarian tumor [3] or an appendiceal mucocele [4], a pre-existing intraperitoneal mucinous neoplasm has been implicated as the primary cause of PMP. As follows, emerging evidence supports the appendiceal rather than ovarian origin of the disease.

### Classification

PMP has been broadly applied to a heterogeneous group of pathological conditions with a similar clinical presentation, with the site of the primary tumor, neoplastic phenotype of the peritoneal tumor cells and classification of the disease being a matter of controversy. However, attempts have been made to better define and classify the condition based on the clinicopathological characteristics. Ronnett et al. [5] suggested a classification of multifocal peritoneal mucinous tumors into three groups. These include disseminated peritoneal adenomucinosis (DPAM), peritoneal mucinous carcinomatosis (PMCA) and a third hybrid group called peritoneal mucinous carcinomatosis with intermediate or discordant features (PMCA I/D), also known as intermediate features group (IFG). According to this clinicopathological classification, DPAM includes histologically benign peritoneal lesions associated with ruptured appendiceal mucinous adenomas as well as those with similar pathology but lacking a demonstrable appendiceal adenoma. Subsequently, Sugarbaker defined PMP as a grade I mucinous adenocarcinoma that arises from an appendiceal adenoma [6]. Later, they stipulated that the term PMP should be exclusively used to describe the clinical syndrome of mucinous ascites accompanied by a characteristic distribution of peritoneal mucinous tumors with the pathologic features of DPAM [7]. Further studies strongly supported the notion that PMP is a generally low-grade, indolent neoplasm of appendiceal origin with rare distant metastases and unlikely involvement of solid organs [1,8,9]. Since neither adenoma nor adenocarcinoma precisely represents the neoplastic nature of PMP, “mucinous neoplasm of low malignant potential” and “low-grade appendiceal mucinous neoplasm” have been proposed in the literature as the alternative terms for pathological description of the PMP tumor [10].

### Pathogenesis

**Figure 1:**
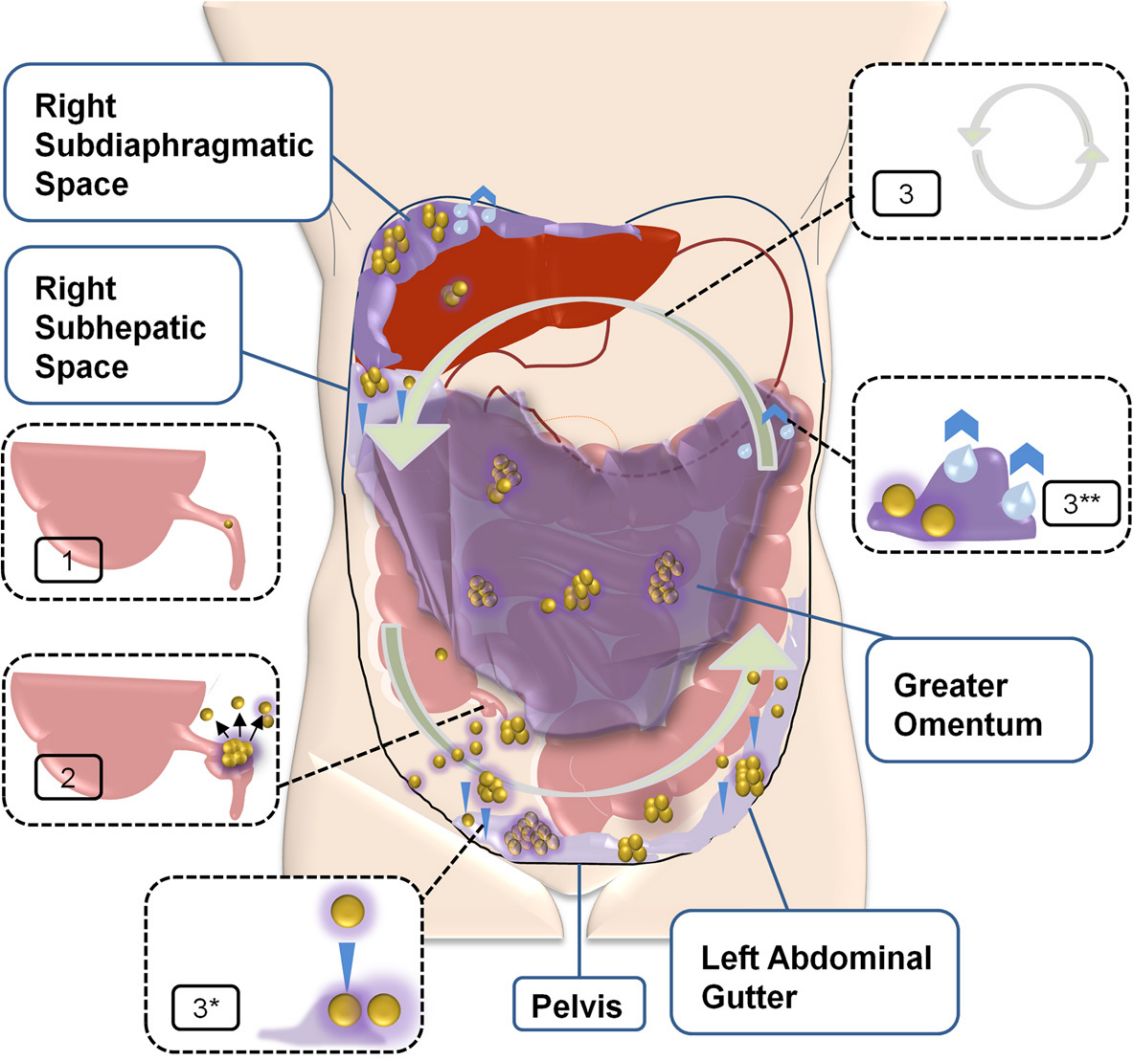
**Schematic representation of the events resulting in the development of PMP.** The pathologic process starts with a neoplastic transformation of the appendiceal goblet cells and development of a primary mucinous tumor (1). Overproduction of mucin and obstruction of the appendiceal lumen lead to the development, and subsequent rupture, of a mucocele (2). Shredded tumor cells gain access to the peritoneal cavity and circulate with the peritoneal fluid (3). Accordingly, tumor cells redistribute and accumulate within the dependent portions of the peritoneal cavity (3*, downward arrows) as well as at the peritoneal fluid reabsorption sites (3**, upward arrows).

The pathological process starts with neoplastic transformation of the appendiceal goblet cells and subsequent formation of a primary mucinous tumor. While proliferating, tumor cells maintain their constitutive level of mucin expression. As a result, the overall secretion of mucin dramatically rises [8]. This is followed by intraluminal accumulation of mucin and eventual development of an appendiceal mucocele. A small perforation or rupture of the mucocele is the key event towards the development of PMP through which tumor cells gain access into the peritoneal cavity. Lacking cell surface adhesion molecules, the exfoliated tumor cells passively circulate with the peritoneal fluid and *redistribute* throughout the peritoneal cavity. As a result, tumor implants and mucin collections form at the peritoneal fluid reabsorption sites as well as within the dependent portions of the peritoneal cavity to create PMP’s characteristic pattern of the peritoneal dissemination (Figure 1) [11]. Accumulating mucin increases intraabdominal pressure and compresses visceral organs. Furthermore, extensive involvement of the peritoneal surface promotes variable inflammatory and fibrotic responses in the peritoneal environment and hence the development of bowel obstruction as a fatal complication of the disease [12,13]. The detailed role of mucin in the pathogenesis of PMP will be discussed later.

### Clinical presentation

The disease is usually diagnosed after the age of 40 [14], with an average age at diagnosis of 53 [15]. PMP clinically presents with a variety of unspecific and sometimes uncommon signs and symptoms. PMP’s clinical manifestations can be roughly classified based on the disease progression (Table 1) [16]. In advanced disease, increased abdominal girth and complaints of abdominal pain related to intestinal obstruction are the most presenting symptom seen in 30-50% of the PMP patients as a result of disseminated mucinous tumor and ascites classically presenting at laparotomy with jelly belly. In less advanced disease, local symptoms are seen in 50-80% of PMP patients without jelly belly ascites and might correspond to the site of the primary tumor, such as appendicitis-like symptoms in 25% of cases, or the location of the peritoneal implants, including lower abdominal pain, pelvic pressure and gynecological complaints in females due to the ovarian deposits of the mucinous tumor in 20-30% of the patients [17,18]. Finally, PMP is coincidentally found in up to 20% of patients undergoing such procedures as laparotomy, laparoscopy or imaging for other medical conditions, e.g. hernia [17-27], bladder tumor [28], jelly like material in urine [29], total uterovaginal prolapse [30], recurrent rectal cancer [31], pregnancy [32] and cesarean section [33]. PMP cases presenting with an ulcerated skin fistula on the right flank [34] or a subcutaneous nontender umbilical nodule [35] have also been reported.

**Table 1:** Common presentations or incidental discovery of PMP on the basis of the disease progression

**Disease status at diagnosis**			**Presenting or incidentally diagnosed with**	
Advanced disease			Abdominal distension, ascites, obstruction [18,36]	
Less-advanced disease	Localized disease		Abdominal pain [18]	Appendicitis-like syndrome
				Presumed cholecystitis
				Vague non-specific pain
				Lower abdominal pain/pelvic mass
	Incidentally diagnosed disease		Gynaecological conditions	Pelvic pain/mass [18,37,38]
		Non-surgical procedures		Infertility investigation [18]
				Postmenopausal bleeding [18,39]
				Abnormal Pap test [18]
			Others [18]	Deep vein thrombosis, rectal bleeding, anaemia
		Laparoscopy or laparotomy [18]	Hernia repair, fibroids, colon cancer, tubal ligation, nephrectomy, abdominal aortic aneurysm repair	

### Diagnosis

#### Imaging

*- Ultrasound and parallel fine needle biopsy*. Ultrasound is accessible and inexpensive. However, conclusions cannot be drawn from ultrasound alone since the mucinous ascites resembles free intraperitoneal fluid [36]. Although cytological investigation of the mucin collections seems to be a useful accompanying procedure, sampling errors, dry taps and false negative results due to low amount of mucin and/or low cellular density are considered as the disadvantages of parallel fine needle biopsy [16].

***-*** *Computed tomography*. Computed tomography (CT) remains the most widely used imaging modality in PMP. Higher densities of mucinous ascites compared to the nonmucinous collections [40], characteristic pattern of the mucinous accumulation [41] and the extent of the disease for preoperative planning and prognostic purposes [36] can be evaluated by CT.

*- Other imaging methods.* Magnetic resonance imaging (MRI) has been described to show the location of mucocele and its morphologic criteria identically to CT. T1- and T2-weighted MRI are more sensitive in distinguishing between mucin and fluid ascites [42-44]. Although positron emission tomography (PET) has been suggested for predicting the peritoneal dissemination [45] and preoperative evaluation of pathological grade and potential for complete cytoreduction [46], its value in PMP remains controversial [47,48].

### Circulating tumor markers

Although relatively non-specific [14], the following tumor markers have been reported to be of value in PMP:

**-***Carcinoembryonic antigen (CEA)*. This tumor marker has been shown to serve as a valuable diagnostic [49] and prognostic tool [49,50] in the management of PMP.

***-*** *Carbohydrate antigen 19.9 (CA19.9).* Practical value of CA19.9 in diagnostic [49] and prognostic [49,51-54] evaluation of PMP has been reported.

**-***Carbohydrate antigen 125 (CA125)* also known as *MUC16.* Although suggested as a marker with diagnostic sensitivity for PMP [52], CA125 is not widely used as a tumor marker for PMP. Instead, as a gynecological tumor marker, it is recommended for exclusion of an ovarian neoplasm [16].

These tumor markers are also used as a baseline value for postoperative follow-up. Moreover, they have been reported as predictors of the completeness of cytoreductive surgery (CRS) [50,52,53,55], a significant prognostic factor for PMP.

### Histopathological analysis

Apart from the characteristic feature of PMP as acellular to paucicellular mucin pools with variable amounts of neoplastic mucinous epithelium, immunohistochemical markers can help to identify the organ of origin. These include positive cytokeratin 20 (CK20), CEA, caudal-type homeobox protein 2 (CDX-2), and MUC2 as well as negative cytokeratin 7 (CK7) and CA125 [16]. Of particular interest is the secreted mucin MUC2 that is extensively positive in the patients. Although MUC2 has been suggested as a biological marker of PMP [56-60], its significance as a prognostic factor is a matter of controversy [61].

### Differential diagnosis

The main entity to be considered in the differential diagnosis of PMP is peritoneal mucinous carcinomatosis arising from a primary mucinous carcinoma. Other differential diagnoses reported in the literature include endometriosis with myxoid change [62], melioidosis (a lethal infectious disease caused by *Burkholderia pseudomallei*) [63] and those with abdominal CT resemblance, e.g. extensive abdominal plexiform neurofibromatosis [64].

### Treatment

Traditional treatment consists of repetitive surgical debulking. Due to the presence of tumor deposits after the first debulking surgery, this approach could results in short term palliation with imminent recurrence or progression, hence redo procedures and a shorter 5- to 10-year overall survival (OS) rate of approximately 50% [65-67]. A more aggressive approach by Sugarbaker [68,69] utilizes peritonectomy and visceral resections, called cytoreductive surgery (CRS), in combination with hyperthermic intraperitoneal chemotherapy (HIPEC) that is featured by direct targeting of the microscopic disease, locoregional drug availability, minimal systemic exposure and improved drug penetration through hyperthermia. This strategy comprises the current standard of care for PMP with known benefits well-documented by us [15,53,54,70-81] and others [82-92]. Other proposed modalities include laparoscopy for less advanced disease [93,94], whole abdominopelvic radiotherapy (WAPRT) as a palliative treatment [95] and use of mucolytic [96-100], antibacterial [58,101,102] and anti-inflammatory [103] agents with potential values as complementary/adjuvant therapies.

### Prognosis

Although PMP as a neoplastic disease runs a chronic, indolent course with late invasion and only rare metastasis outside the peritoneum, it is a morbid, recurrent condition with life-threatening complications. Biological features of the tumor [61,104] and access to the current standard of care at specialized oncology centers with a peritoneal surface malignancy program [105-110] comprise the most important prognostic determinants of PMP. Through a retrospective, multi-institutional study on 2298 patients treated at 16 specialized centers affiliated with the Peritoneal Surface Oncology Group International [15], Chua et al. reported a median survival rate of 196 months (16.3 years) and a median progression-free survival rate of 98 months (8.2 years) as well as 10- and 15-year survival rates of 63% and 59%, respectively.

### Mucin: from intestinal physiology to PMP

#### Mucin family

Mucins are a diverse family of high molecular weight, heavily glycosylated proteins (Table 2). Also known as MUC glycoproteins, mucins are differentially expressed by specialized epithelial cells of mucosal surfaces throughout the body in a relatively organ- and cell type-specific manner [111]. Mucins are categorized into *membrane-associated* and *secreted* types, with the latter being divided to *gel-forming* and *non-gel-forming* subtypes [112,113]. Membrane-associated mucins communicate information about extracellular conditions, mediate intracellular signal transduction, and contribute to morphological and behavioral characteristics of the epithelial cells [114,115].

**Table 2:** Classification, designation and distribution of mucin family

**Type of mucin**		**Designation**	**Site of expression**
**Membrane-associated**		MUC1	Almost all glandular epithelial surfaces of respiratory, gastrointestinal and female reproductive tracts, middle ear, salivary gland, mammary gland and normal pancreatic intralobular ducts
		MUC3A	Gastrointestinal tract epithelium
		MUC3B	Gastrointestinal tract epithelium
		MUC4	Respiratory tract, salivary glands, stomach, colon, eye, vagina, ectocervix, uterus and prostate
		MUC11	Gastrointestinal, respiratory, reproductive and urinary tracts, liver and thymus
		MUC12	Colon, stomach, pancreas, prostate and uterus
		MUC13	Gastrointestinal and respiratory tracts, middle ear and kidney
		MUC15	Placenta, salivary gland, thyroid gland, trachea, esophagus, kidney and testis
		MUC16	Ocular surface, respiratory and female reproductive tracts and middle ear
		MUC17	Gastrointestinal tract, fetal kidney and conjuctival epithelium
		MUC20	Kidney, placenta, lung, prostate, liver, colon, esophagus, rectum and middle ear
		MUC21	Respiratory tract, thymus, colon and testis
**Secreted**	**Gel-forming**	MUC2	Goblet cells of small intestine and colon
		MUC5AC	Tracheobronchial goblet cells, gastric epithelial cells, conjunctiva and lacrimal glands
		MUC5B	Salivary glands, tracheobronchial and esophageal epithelia, pancreatobiliary and endocervical epithelia
		MUC6	Gastric mucosa, duodenal Brunner’s glands, hepatobiliary tract, pancreatic centroacinar cells and duct, basal endometrial and endocervical glands
	**Non-gel-forming**	MUC19	Salivary glands, submucosal gland of the tracheal tissue, corneal and conjunctival epithelia and lacrimal gland tissue
		MUC7	Epithelium of the oral cavity, minor salivary gland, respiratory tract, submucosal glands of the bronchus, conjunctivae and pancreas
		MUC8	Normal Human Nasal epithelial (NHNE) cells and middle ear epithelium
		MUC9	Fallopian tubes (non-ciliated oviductal epithelial cells)

Secreted mucins provide a physical barrier for epithelial cells lining the respiratory and gastrointestinal tracts and form the ductal surfaces of such organs as liver, breast, pancreas and kidney [116]. Moreover, they are part of a defensive system at the mucosal surfaces, including intestinal mucosa.

#### Mucin in intestinal physiology

**Figure 2:**
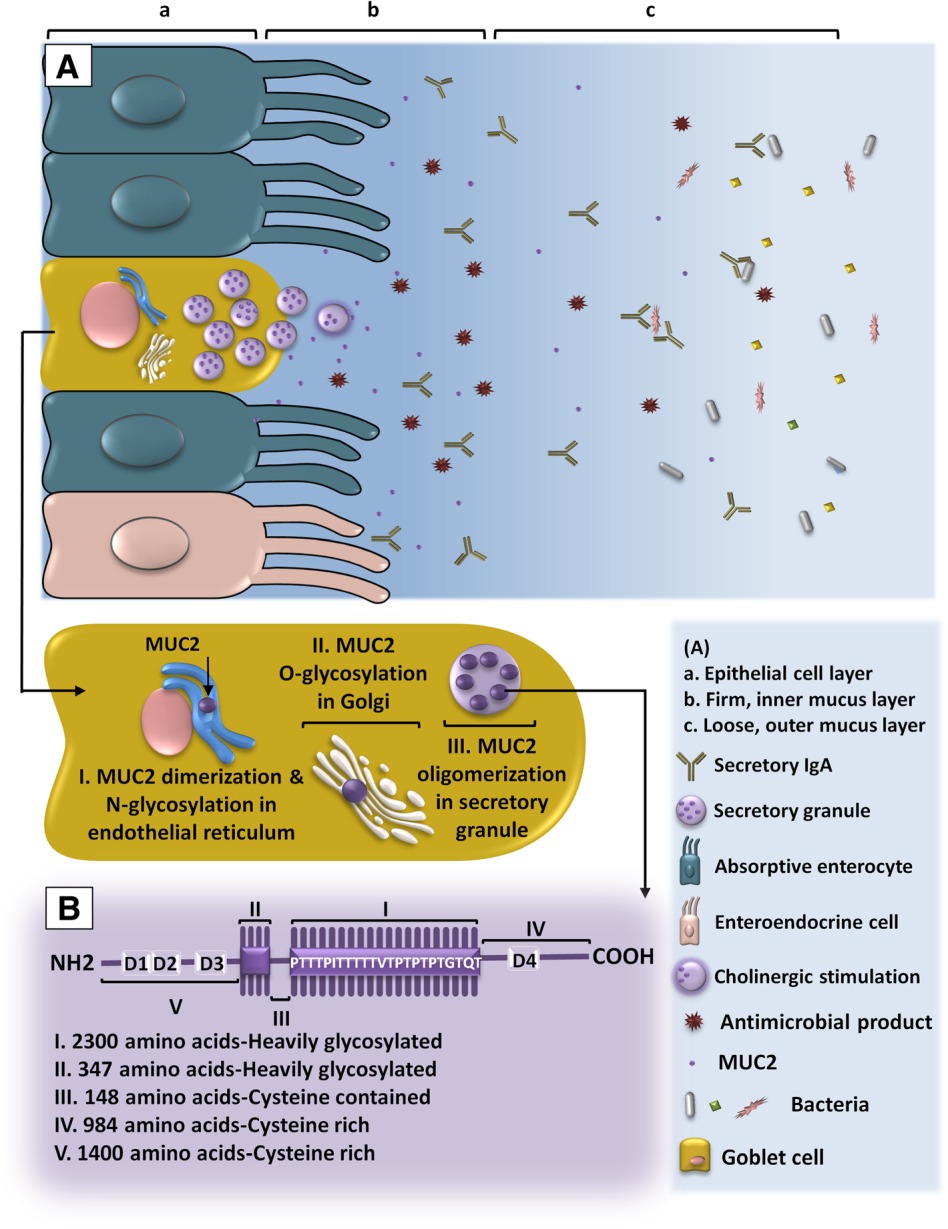
**MUC2 in colonic mucosa. A** Synthesis, secretion and organization of colonic mucosa. The first stage in the biosynthesis of MUC2 is the formation of MUC2 monomer as an N-glycosylated apoprotein in the endoplasmic reticulum. Subsequently, MUC2 dimers are formed when intermolecular disulfide bonds bridge between the C-terminal cysteine knot domains. During transit through the Golgi apparatus, MUC2 dimers become heavily O-glycosylated. Complete glycosylation of the dimers occurs in Golgi where trimerization through disulfide bonds at the N-terminus forms protease-resistant trimers. The fully glycosylated and processed MUC2 is densely packed and stored in secretory granules/vesicles and released through constitutive or stimulated secretory mechanisms. Once released, MUC2 is organized into the firmly adherent inner layer. At a certain distance from the epithelium, this layer is converted into the loose outer layer through proteolytic cleavage and expansion. Mucus also contains immunoglobulins and other proteins. **B** MUC2 structure. The protein core consists of five different regions. Segment (a) and (b) are two central repetitive regions rich in potential O-glycosylation sites, to which branched carbohydrate chains of 4–12 sugars are O-glycosidically linked to form a closely packed sheath around the central protein core. Segment (a), also known as VNTR domain, is a large domain that contains 50–100 “variable number of tandem repeats (VNTRs)” of 23 amino acids, in particular, threonine. Segment (b), also called PTS domain, is a 347 amino acid domain, containing irregular repeats rich in proline, threonine and serine (PTS). These two segments are linked together by segment (c) which is a 148 amino acid, cysteine containing region. Segments (d) and (e) are extensive peptide chains rich in cysteine located at the C and N terminal ends, respectively, containing D domains with sequence homology to von Willebrand factor. These regions are the presumed sites for end to end polymerization of mucin subunits.

While facilitating the transit of intestinal contents [117], secreted mucins participate in the front line of the enteric host defense generated by the alliance of the epithelial cells, immune cells and resident microbiota [118]. This interactive ecosystem is essential for the maintenance of intestinal homeostasis and the normal function and activity of digestive system [119]. The gastrointestinal epithelium and the overlying mucus layer also function as a barrier against intestinal luminal hazards [120]. Colonic mucus is composed of two layers. The outer, loose layer is the habitat of the microbial flora. The inner, dense layer is bacteria-free and firmly attached to the epithelium. This organization keeps the flora well separated from the mucosal surface. The gel-forming mucin MUC2, which is specifically secreted in the small intestine and colon, comprises the substantial component of this double-layered mucus compartment (Figure 2). MUC2 of the inner layer is uncleaved. To form the outer layer, however, MUC2 undergoes proteolytic cleavage to allow expansion of the polymeric structure [121].

#### Mucin in PMP

Under normal conditions, metabolic turnover of intestinal mucin is maintained by the constitutive expression against enzymatic degradation, and, elimination. In PMP, however, mucin is ectopically secreted and increasingly deposited in the peritoneal cavity where it is unable to degrade or drain away. Accumulating mucin causes a major part of the morbidity in PMP. The typical syndrome develops after secreted mucin forms voluminous gels over months and years. Mucin also plays a key role in the biology of the PMP tumor. Most of the tumor cells are surrounded by a mucin coat that allows them to freely move, disseminate and “redistribute” within the peritoneal cavity to create the distinctive feature of PMP. This coating also seems to act as a protective shield against immune recognition and chemotherapy. MUC2, MUC5AC and MUC5B are the gel-forming mucins reportedly found in the PMP secretions. The intestinal mucin MUC2 is known as the PMP-specific mucin. According to O’Connell et al. [8,56], primary ovarian mucinous tumors essentially express MUC5AC whereas solitary appendiceal mucinous tumors and different categories of PMP express MUC2 along with MUC5AC. This finding also supports the notion that PMP is a neoplasm of appendiceal origin. In their studies, O’Connell et al. also showed that MUC2 is behind the high degree of gelation formed in PMP. Since MUC2 is more extensively glycosylated, it is more voluminous than MUC5AC on an equimolar basis, hence formation of abundant mucinous collections where the average mucin:cell ratio is higher than 10:1. Taken together, the investigators concluded that PMP is a disease of the MUC2-secreting goblet cells and that MUC2 could serve as a molecular marker for PMP [8,56]. Since the expression level of MUC2 in PMP is seemingly independent of the degree of the malignant transformation, prognostic significance of MUC2 is controversial [61].

In two case studies, Mall et al. reported the presence of MUC5B, in addition to MUC2 and MUC5AC, in the PMP material [122,123]. Based on the investigations by Sheehan et al. implicating a low-charge glycoform of MUC5B in the production of a tenacious respiratory mucus plug [124,125], Mall et al. speculated that it may be MUC5B that is responsible for the semisolid material found in some PMP patients. Given the high protein content of the PMP secretions, they also raised the possibility that interactions between mucin and non-mucin proteins could contribute to the viscous nature of the PMP exudates [122,123]. Table 3 summarizes a number of studies in which disease-specific expression pattern of MUC2 and other mucins in PMP has been explored.

**Table 3:** Some studies exploring the expression of MUC2 against other mucins in PMP (2002–2012)

**Study**	**Year**	**Number of PMP cases**	**Percentage of cases exhibiting the expression of mucins**	
			**MUC2**	**Other forms of mucins**
O’Connell et al. [8]	2002	100	98%	MUC5AC 95%
O’Connell et al. [56]	2002	25	96%	MUC5AC 92%
Mohamed et al. [126]	2004	33	97%	MUC1 57.5%
Kinkor et al. [127]*	2005	3	?	-
Nonaka et al. [128]	2006	42	100%	MUC5AC 100%
Mall et al. [122]	2007	1	100%	MUC5AC 100%
				MUC5B 100%
Ferreira et al. [129]	2008	7	100%	MUC1 28.6%
				MUC5AC 100%
				MUC6 28.6%
Semino-Mora et al. [58]**	2008	16	N/A^‡^	N/A^‡‡^
Baratti et al. [61]***	2009	85	100%	MUC5AC 87.5%
Flatmark et al. [59]	2010	5	100%	MUC1 0%
				MUC5AC 40%
				MUC4 100%
Guo et al. [60]	2011	35	94.3%	MUC1 0%
Mall et al. [123]	2011	1	100%	MUC1 0%
				MUC4 100%
				MUC5AC 100%
				MUC5B 100%
				MUC6 0%
Chang et al. [130]****	2012	4	64%†	MUC5AC 43%††

*Full-text article, in Czech, not accessible. Results of IHC study not available.

**This study reports the expression of MUC2 and MUC5A as the volumetric density of apomucin (Vvi/10^4^ μm) in such compartments of DPAM and PMCA tissues as epithelium, lymphoid aggregates, stroma vessels and free mucin, respectively, as follows:

‡MUC2: in DPAM: 264 ± 60, 47 ± 16, 31 ± 14 and 261 ± 51; in PMCA: 356 ± 90, 170 ± 26, 117 ± 25 and 1043 ± 282.

‡‡MUC5AC: in DPAM: 90 ± 13, 345 ± 20, 65 ± 17, 37 ± 6; in PMCA: 56 ± 12, 246 ± 17, 50 ± 15 and 48 ± 9.

***The percentages shown for this study are numerical estimations of data originally presented in a column graph.

****In this study, among a total of 14 patients with mucinous adenocarcinoma, 4 cases have reportedly exhibited PMP syndrome. Results of the expression of MUC2 and MUC5AC, however, are reported in total, with no data individually available regarding the PMP cases.

†, ††Data shown is the percentage of MUC2/MUC5AC expression in all patients with mucinous adenocarcinoma, including PMP ones.

#### Mucin elimination in the management of PMP

Mucin comprises the cornerstone of the PMP pathogenesis. However, optimal removal of mucin and mucinous implants via conventional therapies remains challenging, with the residual disease accounting for the recurrence of the condition. In order to enhance the current standard of care, novel strategies are required to more effectively eliminate mucin and its source. As such, some efforts have been made to disrupt PMP production of MUC2. In this regard, targeting biological mechanisms regulating the mucin synthesis at transcriptional and post-transcriptional levels could be of potential value. Since MAPK pathway has been implicated in the pathological induction of MUC2 by *Pseudomonas aeruginosa* infection [131] and mucoepidermoid carcinogenesis [132], targeted inhibition of MAPK might be of therapeutic benefit in PMP. On the basis of the cross-talk between MAPK and other inflammation-associated signaling pathways [133], multi-targeted agents may be more effective in PMP where the disease develops within an inflammatory milieu [134].

In addition, PMP inflammatory microenvironment with a unique profile of cytokines [13] contributes to the mucin overproduction. Cytokines reportedly upregulate the expression of *MUC2* and enhance the mucin secretion [135-137]. Glucocorticoids, on the other hand, have been shown to downregulate *MUC2* via direct inhibition of glucocorticoid response elements (GREs) and indirect transrepression of inflammation-associated transcription factors [138,139]. Choudry et al. recently demonstrated dexamethasone- and celecoxib-induced inhibition of the mucin production in a mucin-secreting cancer cell line with goblet cell phenotype as well as in a murine model of PMP [103]. Methylation of the *MUC2* promoter has also been suggested as a potential tool for manipulating the expression of MUC2. Okudaira et al. showed that colorectal cancer (CRC) cell lines of mucinous type exhibit low-level methylation at the *MUC2* promoter as compared to non-mucinous cell lines. They concluded that low methylation status of the *MUC2* gene plays a predominant role in the high level MUC2 expression in mucinous CRC [140]. Through inhibiting the *MUC2* promoter methylation, the Sp-family of transcription factors augments MUC2 expression. Thus, mithramycin, a Sp1 binding site inhibitor, effectively blocks the MUC2 expression in colorectal cancer [132,141].

Breakdown of mucin as well as enhancement of cytoreduction through locoregional therapies outlines our intended approach to this challenge. In this regard, we aim to develop a novel treatment for facilitated, enhanced removal of the mucin-tumor burden at both macroscopic and microscopic levels. For this purpose, a panel of mucin-secreting cancer cells of gastrointestinal and peritoneal origin and animal models of PMP [142] and peritoneal carcinomatosis [143] are employed in our preclinical investigations. Among a number of mucolytic agents studied [99,100], bromelain and N-acetyl cysteine (NAC), two generally-safe mucolytics of plant sources [144,145], have shown promise. Our preliminary results indicate bromelain- [146-148] and NAC-induced inhibition of the growth and proliferation of the mucin-producing cancer cells *in vitro*, with the cytotoxicity being augmented when the two are used in combination (unpublished data). We have also observed the ability of these mucolytics in dissolving both the mucin samples from PMP patients *ex vivo* and the mucin implants in a xenograft model of PMP *in vivo* with no treatment-related toxicity in rats [149]. We are currently performing final preclinical testing of our mucolytic compound before proceeding to the clinical phase.

## Conclusion

Despite its morbid, debilitating nature with severe impact on quality of life, PMP remains orphan and enigmatic. Essential for physiological function of the gastrointestinal tract, mucin is the major contributor to the pathophysiology of PMP. Peritoneal implantation of the tumor cells originating from an appendiceal mucinous tumor results in the progressive accumulation of ectopic mucin. Given the clinicopathological profile of the disease compatible with an indolent, generally low grade malignancy, multifocal collections of voluminous mucin and the ensuing complications are the main determinants of the disease prognosis. As the predominant, gel-forming mucin secreted in PMP, MUC2 is responsible for the high degree of gelation and the characteristic feature of the clinical syndrome. Despite the current standard of care as extensive surgical resection combined with chemotherapy, PMP frequently recurs; with treatmen options being limited at recurrence. On this basis, in-depth investigations are warranted to illuminate unknown aspects of the disease and to seek novel therapeutic approaches for an enhanced treatment. Owing to the substantial role of mucin in the pathogenesis of PMP, development of strategies for targeting mucin and its biology seems to be of particular significance that needs to be further explored in future studies.

## Abbreviations

CA125: Carbohydrate antigen 125; CA19.9: Carbohydrate antigen 19.9; CDX-2: Caudal-type homeobox protein 2; CEA: Carcinoembryonic antigen; CK7: Cytokeratin 7; CK20: Cytokeratin 20; CRC: Colorectal cancer; CRS: Cytoreductive surgery; CT: Computed tomography; DPAM: Disseminated peritoneal adenomucinosis; GREs: Glucocorticoid response elements; HIPEC: Hyperthermic intraperitoneal chemotherapy; IFG: Intermediate features group; MRI: Magnetic resonance imaging; NAC: N-acetyl cysteine; NORD: National organization for rare disorders; ORDR: NIH office of rare diseases research; PET: Positron emission tomography; PMCA: Peritoneal mucinous carcinomatosis; PMCA I/D: Peritoneal mucinous carcinomatosis with intermediate or discordant features; PMP: Pseudomyxoma peritonei; WAPRT: Whole abdominopelvic radiotherapy.

## Competing interests

The authors declare that they have no competing interests.

## Authors’ contributions

AA designed and performed the systematic literature search supervised by DLM. AA drafted and edited the manuscript. SMM and AE reviewed the initial draft. SMM designed and prepared the figures. DLM critically assessed the initial draft. All authors read and approved the final manuscript.
